# Development of Kinematic 3D Laser Scanning System for Indoor Mapping and As-Built BIM Using Constrained SLAM

**DOI:** 10.3390/s151026430

**Published:** 2015-10-16

**Authors:** Jaehoon Jung, Sanghyun Yoon, Sungha Ju, Joon Heo

**Affiliations:** School of Civil and Environmental Engineering, College of Engineering, Yonsei University, 50 Yonsei-ro, Seodaemun-gu, Seoul 120-749, Korea; E-Mails: lionheart_kr@yonsei.ac.kr (J.J.); yoonssa@yonsei.ac.kr (S.Y.); jsh4907@yonsei.ac.kr (S.J.)

**Keywords:** SLAM, laser scanner, point clouds, line feature, constrained least squares adjustment

## Abstract

The growing interest and use of indoor mapping is driving a demand for improved data-acquisition facility, efficiency and productivity in the era of the Building Information Model (BIM). The conventional static laser scanning method suffers from some limitations on its operability in complex indoor environments, due to the presence of occlusions. Full scanning of indoor spaces without loss of information requires that surveyors change the scanner position many times, which incurs extra work for registration of each scanned point cloud. Alternatively, a kinematic 3D laser scanning system, proposed herein, uses line-feature-based Simultaneous Localization and Mapping (SLAM) technique for continuous mapping. Moreover, to reduce the uncertainty of line-feature extraction, we incorporated constrained adjustment based on an assumption made with respect to typical indoor environments: that the main structures are formed of parallel or orthogonal line features. The superiority of the proposed constrained adjustment is its reduction for uncertainties of the adjusted lines, leading to successful data association process. In the present study, kinematic scanning with and without constrained adjustment were comparatively evaluated in two test sites, and the results confirmed the effectiveness of the proposed system. The accuracy of the 3D mapping result was additionally evaluated by comparison with the reference points acquired by a total station: the Euclidean average distance error was 0.034 m for the seminar room and 0.043 m for the corridor, which satisfied the error tolerance for point cloud acquisition (0.051 m) according to the guidelines of the General Services Administration for BIM accuracy.

## 1. Introduction

A Building Information Model (BIM) is based on 3D models that organize and represent as-designed construction site information, whereas as-built information usually is derived from monitoring activities. Comparison of as-designed with as-built information facilitates quality control and enhances building management efficiency [1]. Recently introduced 3D laser scanners make possible rapid and accurate capturing of a huge number of point clouds, which produces very dense and elaborate coordinate data points for the surfaces of a physical object [2,3]. Integration of laser scanning with BIM can yield significant advantages over traditional approaches, specifically by facilitating fast and accurate data acquisition for existing conditions [4,5,6,7]. In the AEC (Architecture, Engineering, and Construction) domain, correspondingly, the 3D “as-built BIM” has become an essential means of accurately representing recently constructed buildings and their facilities to support maintenance process [8,9]. The major focus in this study is the development of efficient 3D data acquisition system for input of as-built BIM creation.

Conventional static laser scanners capture data from objects in their line of sight. Ensuring a complete map in the presence of occlusions necessitates scans from multiple positions, which result in a number of point cloud groups. The process of transforming multiple point clouds into a single point cloud is called registration. Registration of multiple point clouds requires that surveyors setup the laser scanner at a position with known coordinates or position artifacts (known as targets) in the overlap areas. Not surprisingly, using targets to merge multiple point clouds incurs additional cost and time in scanning-position surveying and manual post-processing. Moreover, it requires accurate instrument installation; any error at any given position renders the data collected there unusable [10,11,12]. In any case, indoor mapping applications involving very complex office environments with many occlusions certainly impose severe operational limitations on conventional static scanning systems.

Alternatively, we propose herein a kinematic 3D laser scanning system that continuously scans and registers point cloud data using feature-based Simultaneous Localization And Mapping (feature-based SLAM) technique. The feature-based SLAM has been employed for autonomously navigating mobile systems with 2D laser scanner that horizontally map the surrounding environment and use the acquired features for system-position correction. One way to acquire a 3D map is to use an additional scanner to scan the vertical profiles of the environment along the system’s trajectory. In this case, the accuracy of 3D data depends on that of the system’s position [13,14,15,16,17]. Unfortunately however, the feature-based SLAM suffers from data association errors due to incorrect extraction and matching of feature extractions [18].

In order to improve the performance of feature-based SLAM, constraint approaches are interesting solutions that modify the basic algorithm according to some environmental assumptions. This allows, for all cases that do not violate those assumptions, much improved performance [19,20]. The basic assumptions, specifically for indoor environments, are as follows: (1) the main structures (e.g., walls and doors) are formed of straight lines; (2) all such structures are parallel or perpendicular to each other. Zunino [21] used the orientation of the first-extracted line as a reference angle and corrected the other lines to fulfill the 90° geometric constraint. Nguyen *et al.* [22] suggested orthogonal SLAM, by which only lines that are parallel or perpendicular to each other are mapped. Using the lengths of line segments as weights, they defined the reference line segment for horizontal and vertical directions and rotated the other lines around their midpoints to align them with the reference orientation; finally, the system’s position and its surrounding map were updated according to the orientations and relative distances between the corrected lines. The main drawback of the above methods is that they are basically heuristic approaches: they do not statistically consider observation (point cloud) errors in correcting extracted lines with respect to geometric constraints. Choi *et al.* [23] aligned the first extracted line parallel with x-axis and compared its colinearity and geometric constraints with the other lines. If a line satisfied both conditions, it was merged with the first line; if it satisfied only the geometric constraints, it was added as a new feature. However, at the starting point, they always need to align the system’s initial direction with the main structure of the environment and update a single line segment separately without considering its geometric relationships with the other lines. Kuo *et al.* [24] incorporated the orthogonal assumptions into the lightweight Rao-Blackwellized Particle Filter (RBPF) SLAM. They picked up a reference line that has been observed most of the time and identified whether the other lines are orthogonal to the reference one. By filtering out the non-orthogonal lines, they could increase the accuracy and reduce the complexity when calculating the importance weight of each particle in RBPF process. However, they did not use the orthogonal constraint to adjust the line parameters. Recently, Choi, *et al.* [25] proposed a soft constrained SLAM system that utilizes a monocular upward-looking camera. The camera extracts line and point features on the ceiling: both are detected repeatedly and consistently for long periods of operation time. The distances between line and point measurements are calculated and applied in the constrained Extended Kalman Filter (EKF) framework. Since the constraint is not derived from a priori knowledge but rather from an observed geometric relationship, it is considered a soft constraint method. Nonetheless, further studies are necessary to exploit the soft constraint approach for laser-scanning-based SLAM.

This research formulated a new feature-based SLAM technique incorporating a constrained least squares method. The superiority of the proposed approach, compared with the previous works, lies in its direct adjustment of extracted line features according to the parallel or orthogonal conditions: the least squares method accounts for the presence of errors in point cloud observations and decreases the uncertainties of estimations of final line-feature parameters [26,27], which leads to successful data association. For the proposed approach, the Unscented Kalman Filter (UKF) algorithm was chosen, because it is a widely used means of estimation for feature-based SLAM and is easy to implement [28,29]. The performance of the proposed approach was tested both with and without the constrained adjustment. The accuracy of the constrained kinematic 3D laser scanning system’s point cloud acquisition was evaluated by comparison with the measurements acquired by a total station. Additionally, to investigate the feasibility of the point cloud acquisition in BIM perspective, further evaluation was performed in reference to the guidelines of the General Services Administration for BIM accuracy [30].

## 2. Methods

### 2.1. Overview

Features contain both semantic and metric information: semantically, they provide the feature type such as point, line or plane; and metrically they provide geometric parameters such as range and orientation [19,31]. The feature-based SLAM technique entails incrementally building a map of features in the environments and using this feature map to simultaneously localize the mobile system [20]. In case of line-feature-based SLAM, the basic assumption, particularly for building interiors, is that the physical structures can be modeled by a set of orthogonal or parallel lines, though this requires a reliable feature extraction technique [32,33].

**Figure 1:**
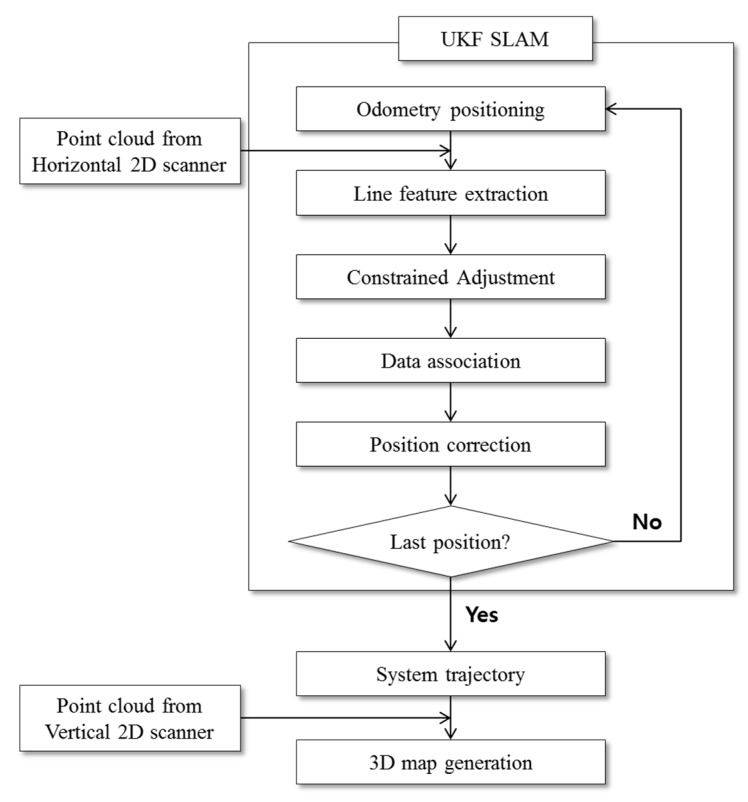
Study flow chart.

This research proposes a constrained least squares method that adjusts the extracted line features according to the geometric conditions (orthogonality or parallelism) to effect better localization quality. Figure 1 shows the overall process of the proposed approach. The current mobile system, which has two wheels on the left and the right, initially predicts its state based on odometry information, but it is strongly influenced by the accumulation of errors, which results in considerable location errors at the end [24]. Assuming the line features taken from scans to be more reliable, they can be used to correct the system’s state through data association. Unfortunately, uncertainties arising from scan data can lead to incorrect feature extraction and failure in data association step [18,34]. The proposed constrained approach is applied to adjust the line-feature extractions, which helps to reduce uncertainties, thus leading to successful data association. Finally, based on the corrected system’s locations, 2D vertical point profiles are sequentially registered. In this way, the 3D environment is reconstructed along the system’s trajectory.

### 2.2. Odometry Positioning

The Kalman Filter based SLAM estimates a process state at some given time and then obtains feedback in the form of measurements. As such, its equations fall into two groups: control update equations (the state model) and measurement update equations (the measurement model). The state model is responsible for projecting forward (in time) the current state and error covariance estimates to obtain the *a priori* estimates for the next time step. The measurement model is responsible for the feedback; *i.e*., for the incorporation of a new measurement into the a priori estimate to obtain an improved *a posteriori* estimate [35].

In the control update, provided that the state estimation of the current kinematic scanning system (position and orientation) at time *t* is $\hat{\text{X}}(t)={\left[\begin{array}{ccc} x(t) & y(t) & \theta (t) \end{array}\right]}^{\text{T}}$, the next state ${\hat{\text{X}}}^{-}(t+1)$, which is the displacement of the mobile system between two intermediate points along its trajectory, can be obtained by odometry dead-reckoning. The odometry model relies on a piecewise approximation using the displacements of the left and the right wheel as

(1) $$O={\left[\begin{array}{cc} s_{r} & s_{l} \end{array}\right]}^{\text{T}}$$

Aiming to estimate the *a priori* state of the kinematic scanning system at time *t* + 1, the following transition function is used:

(2) $${\hat{\text{X}}}^{-}(t+1)=f(\hat{\text{X}}(t),O(t))$$

(3) $$\;\;=\;\left[\begin{array}{c} x(t)+s\cdot \cos(\theta (t)+\Delta \theta /2)\\ y(t)+s\cdot \sin(\theta (t)+\Delta \theta /2)\\ \theta (t)+\Delta \theta \end{array}\right]$$

(4) $$=\left[\begin{array}{c} x(t)+(s_{r}+s_{\ell })/2\cdot \cos(\theta (t)+\left(s_{r}-s_{\ell }\right)/2b)\\ y(t)+(s_{r}+s_{\ell })/2\cdot \sin(\theta (t)+\left(s_{r}-s_{\ell }\right)/2b)\\ \theta (t)+\left(s_{r}-s_{\ell }\right)/b \end{array}\right]$$

where *s* and ∆θ represent the distance and angular displacement respectively between two consecutive time steps *t* and *t* + 1, and *b* is the baseline between two wheels [36].

### 2.3. Line-Feature Extraction

After the displacement, only the location of the mobile system changes, as estimated by odometry, while the locations of map features, being static entities, remain the same as estimated in the previous time instant. Since the odometry information is often erroneous, we cannot rely directly on it [37], but we can use the map features of the environment to estimate the *a posteriori* state of the kinematic scanning system because the displacement of the mobile system produces changes in the dependencies existing between the location of the mobile system and those of the map features [38]. This is accomplished by scanning the features from the surrounding environment and re-observing them while the system moves around [39]. The line segment, as represented by the Hessian model, is the commonly employed feature in SLAM [40,41]:

(5) xcosϕ+ysinϕ−ρ=0

where the line parameters $Y={\left[\begin{array}{cc} \phi & \rho \end{array}\right]}^{\text{T}}$ are the orientation and distance from the origin, respectively.

**Figure 2:**
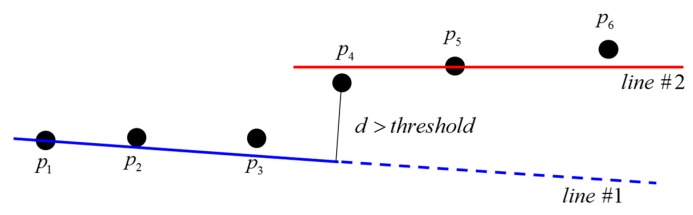
Incremental line extraction algorithm (adapted from [43]).

In the present study, an incremental algorithm is used to extract line features from the laser scan data, owing to its superior speed and correctness compared to other line-extraction algorithms [42]. The incremental algorithm starts with the first two points (*p*_1_ and *p*_2_ in Figure 2) to construct a line. It then adds the next point to the current line model (*line* #1 in Figure 2), and re-computes the line parameters. If a predefined condition (e.g., the variances of line parameters) is satisfied, it continues to add new points (*p*_3_–*p*_6_ in Figure 2); otherwise, it puts back the last point and computes new line parameters (*line* #2 in Figure 2) [42,43].

### 2.4. Constrained Adjustment

**Figure 3:**
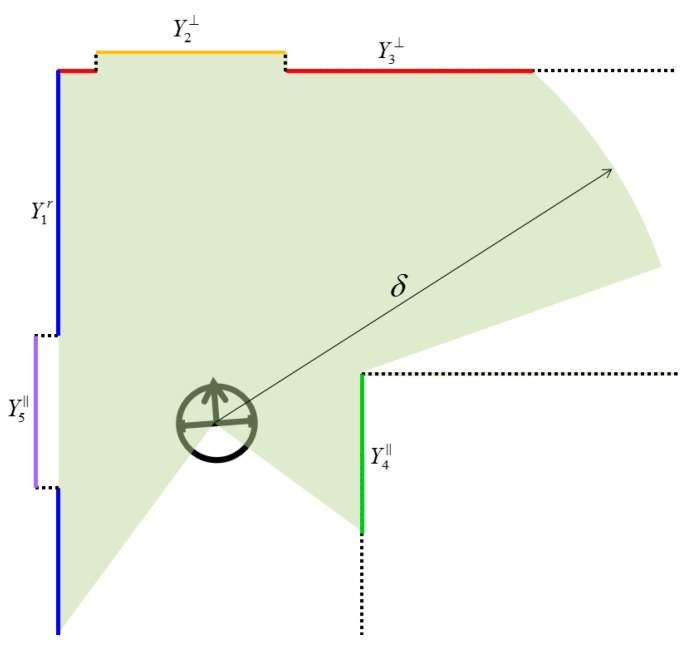
Constrained relations of extracted line features.

The proposed constrained approach is based on the fact that in most indoor environments, major structures, such as walls and doors, can be represented by sets of lines that are orthogonal or parallel to each other. Assuming that the first line *Y^r^_1_* is the reference, the conditional equations for the other orthogonal or parallel lines *Y_i_* are defined as

(6) $$if\;|\phi_{1}^{r}-\phi_{i}|-\pi /2<\theta \;,\;\;Y_{i}\;\;\text{is}\;\;\text{orthogonal}\;\;(⊥)\;\;\;\;\;\;i=2,\cdots ,n$$

(7) $$if\;|\phi_{1}^{r}-\phi_{i}|-\pi <\theta \;\;or\;\;|\phi_{1}^{r}-\phi_{i}|<\theta \;\;,\;\;Y_{i}\;\;\text{is}\;\;\text{parallel}\;\;(∥)\;\;\;\;\;i=2,\cdots ,n$$

where $\phi_{1}^{r}$ and ϕ*_i_* are the orientation of the reference and the other line, respectively (ranging from −π to π), θ is the threshold to identify the orthogonal lines: θ = 10° was empirically determined because it effectively filtered out the arbitrarily-oriented lines while reserving the lines which are slightly off the constraints (possibly due to sensor imperfections) in this research. In Equation (7), the former condition indicates the case that the other line *Y_i_* is located in the same side as the reference line *Y^r^_1_*, and the latter condition, vice versa. Figure 3 illustrates the conceptual idea of the orthogonal and the parallel relationships of extracted line features. As indicated in the figure, a total of five lines are detected within the range (δ) of the laser scanner. Among them, a line including the largest number of point clouds is selected as the reference line (*Y^r^_1_*). If the other lines satisfy conditional Equation (6), they are considered to be orthogonal, as $Y_{2}^{⊥}$ and $Y_{3}^{⊥}$; otherwise, if the other lines are on the opposite side of the reference line and satisfy the former condition of Equation (7), or if they are on the same side of the reference line and satisfy the latter condition of Equation (7), they are considered to be parallel, as $Y_{4}^{∥}$ and $Y_{5}^{∥}$, respectively.

To formulate the matrix expression, the normal matrix and its matching constraints matrix are formed. In this procedure, the constraint equations border the normal equations as

(8) $$\left[\begin{array}{cc} J^{\text{T}}WJ & Z^{\text{T}}\\ Z & 0 \end{array}\right]\left[\begin{array}{c} \hat{\xi }\\ \hat{\lambda } \end{array}\right]=\left[\begin{array}{c} J^{\text{T}}W\tau \\ \tau_{c} \end{array}\right]$$

(9) $$f_{i}=x\cos\phi_{i}+y\sin\phi_{i}-\rho_{i}$$

(10) Jξ^=τ, J=[A1A2⋱Ai], Ai=[∂fi1∂ϕi∂fi1∂ρi∂fi2∂ϕi⋮∂fini∂ϕi∂fi2∂ρi⋮∂fini∂ρi], ξ^=[Δϕ1Δρ1Δϕ2Δρ2⋮ΔϕiΔρi], τ=[−f1(Y10)1⋮−f1(Y10)n1⋮−fi(Yi0)1⋮−fi(Yi0)ni]

**Figure 4:**
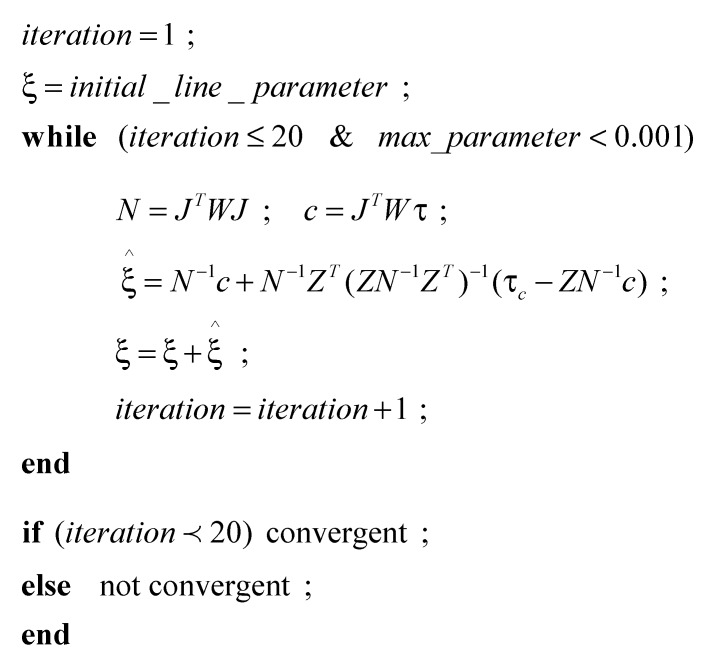
Iterative process of nonlinear least squares adjustment with constraints.

The Equation (10) is a linearization of Equation (9) by Taylor series, where *J* is the Jacobian matrix of the Hessian line model Equation (9) with respect to ϕ and ρ, τ is the observed minus computed values, $\hat{\xi }$ is correction values of line parameters, *Y_i0_* is initial approximation of line parameters (ϕ*_io_* ρ*_io_*) of line *i. A_i_* indicates the matrix of partial differentials, where *i* is number of lines and *n_i_* is number of points which was used to extract the line. Correspondingly, the Jacobian matrix of the constraint model (Equations (6) and (7)) is formed with respect to $\phi_{1}^{r}$ and ϕ*_i_* then included in the normal matrix as additional rows *Z* and columns *Z^T^*, and their constants ±π2   or ±π are added to the constants matrix as additional rows τ*_c_*. For example, in the case of Figure 3, line $Y_{2}^{⊥}$ and $Y_{3}^{⊥}$ satisfy the orthogonal condition, and line $Y_{4}^{∥}$ and $Y_{5}^{∥}$ satisfy the parallel condition relative to the reference line (*Y^r^_1_*). Therefore the matrix form of the constraint equation is formed as shown in Equation (11) and the first term of Equation (11) will be matrix *Z*. The signs in front of π2 and π are dependent on the angle parameter difference, positive or negative. Weight matrix (*W*) is based on the number of points which was used to extract a line (Equation (12)). τ is the observed minus computed values. $\hat{\xi }$ is the estimated correction value for the line-feature parameters while $\hat{\lambda }$ is the additional row for Lagrangian multipliers.

(11) $$Z\hat{\xi }=\tau_{c},\;Z=\left[\begin{array}{cccc} 1 & 0 & -1 & 0\\ 1 & 0 & 0 & 0\\ 1 & 0 & 0 & 0\\ 1 & 0 & 0 & 0 \end{array}\begin{array}{cccc} 0 & 0 & 0 & 0\\ -1 & 0 & 0 & 0\\ 0 & 0 & -1 & 0\\ 0 & 0 & 0 & 0 \end{array}\begin{array}{c} 0\\ 0\\ 0\\ -1 \end{array}\begin{array}{c} 0\\ 0\\ 0\\ 0 \end{array}\right],\;\hat{\xi }=\left[\begin{array}{c} \begin{array}{l} \Delta \phi_{1}\\ \Delta \rho_{1}\\ \Delta \phi_{2} \end{array}\\ \begin{array}{c} \Delta \rho_{2}\\ \begin{array}{l} \Delta \phi_{3}\\ \Delta \rho_{3} \end{array}\\ \begin{array}{l} \Delta \phi_{4}\\ \Delta \rho_{4} \end{array} \end{array}\\ \begin{array}{l} \Delta \phi_{5}\\ \Delta \rho_{5} \end{array} \end{array}\right],\;\tau_{c}=\left[\begin{array}{c} \frac{\pi }{2}\\ \frac{\pi }{2}\\ \pi \\ -\pi \end{array}\right]$$

(12) $$W={\left[\begin{array}{cccc} W_{1} & & & \\ & W_{2} & & \\ & & \ddots & \\ & & & W_{i} \end{array}\right]}_{\left(N\;\text{X}\;N\right)},\;W_{i}={\left[\begin{array}{ccc} \frac{n_{i}}{N} & & \\ & \ddots & \\ & & \frac{n_{i}}{N} \end{array}\right]}_{\left(n_{i}\;x\;n_{i}\right)}$$

where, *n_i_* is number of points when extracting *i*th line and *i* is number of lines extracted. Finally, the correction $\hat{\xi }$ and the dispersion $\text{D}\{\hat{\xi }\}$ (the adjusted covariance) of the line parameters are calculated as

(13) $$\hat{\xi }=N^{-1}c+N^{-1}Z^{\text{T}}{\left(ZN^{-1}Z^{\text{T}}\right)}^{-1}\left(\tau_{c}-ZN^{-1}c\right)$$

(14) $$\text{D}\{\hat{\xi }\}=\sigma_{0}^{2}\left\{N^{-1}-N^{-1}Z^{\text{T}}{\left(ZN^{-1}Z^{\text{T}}\right)}^{-1}ZN^{-1}\right\}$$

where $\sigma_{0}^{2}$ is the reference variance, *N* indicates the normal matrix *J^T^WJ*, and *c* is *J^T^W*τ. For nonlinear least squares adjustment, the initial value is necessary. The extracted line parameter from the point cloud data was used as initial value. The initial value is continuously adjusted by the computed values which were based on the constraint conditions. The adjustment process is repeated until the computed value ($\hat{\xi }$) become sufficiently small [26,27,44]. In this study, process is repeated until the total sum of the line parameter’s increment ($\hat{\xi }$) gets smaller than 0.001 or the number of iteration reaches 20 times. For the distance (ρ) value the threshold 0.001 means 0.001 m, and for the orientation (ϕ) it means 0.001 rad which results in 0.0017 m error per 10 m. Figure 4 shows the detail process. The number of iteration (≤20) was checked as a convergent condition. When the iteration hits 20 times, it is not considered to be convergent, and the line is eliminated from the line list. Note that both $N^{-1}$ and $Z^{\text{T}}Z$ in Equation (14) are symmetric, positive definite matrix, the constraints will correspondingly decrease the uncertainties of adjusted parameters.

The most important aspect of least squares adjustment, for which reason it is superior to the other heuristic approaches for constrained SLAM, is its permission of all point observations with corresponding Jacobian matrix (*J*) and constraint condition matrix (*Z*) to be entered into a single adjustment equation and used simultaneously in the computations. The adjusted line parameters satisfy the geometric constraints (orthogonality or parallelism) and minimize the weighted residuals under the given constraints [26], thus resulting in better consistency between line extractions at different time steps for the next process, data association.

### 2.5. Data Association

Once the mobile system obtains sensor readings at any position, a way of paring the newly observed features to the past observations has to be defined. It is called data association and plays an important role because the system’s pose can be well estimated only when data association is correct [45]. New features at time *t* + 1 are obtained from horizontal scanning in the body frame (b), whereas previous features until time t are stored in the global frame (g), thus the transformation model to the corresponding sensor is defined as

(15) $$\begin{array}{l} {\hat{\phi }}_{b}(t+1)=\phi_{g}(t)-{\hat{\theta }}_{g}(t+1)\\ {\hat{\rho }}_{b}(t+1)=\rho_{g}(t)-{\hat{x}}_{g}(t+1)\cos\phi_{g}(t)-{\hat{y}}_{g}(t+1)\sin\phi_{g}(t) \end{array}$$

where $\phi_{g}(t)$ and $\rho_{g}(t)$ are the orientation and distance of the previously observed features $Y_{g}(t)$, and ${\hat{\phi }}_{b}(t+1)$ and ${\hat{\rho }}_{b}(t+1)$ are the parameters of the predicted features ${\hat{Y}}_{b}(t+1)$ with respect to the a priori state ${\hat{X}}^{-}(t+1)$ obtained by Equations (2)–(4). The pairing process is performed by comparing every possible newly observed line features $Y_{b}(t+1)$ with predicted line features ${\hat{Y}}_{b}(t+1)$ using Mahalanobis distance defined as

(16) $$\begin{array}{l} \chi_{\gamma ,d}^{2}\geq {\left(Y_{b}^{i}-{\hat{Y}}_{b}^{j}\right)}^{\text{T}}{\left(C^{i}+C^{j}\right)}^{-1}(Y_{b}^{i}-{\hat{Y}}_{b}^{j})\;\\ \;\;\;\;\;\;i=1,\cdots ,n\;\\ \;\;\;\;\;j=1,\cdots ,m \end{array}$$

where *n* and *m* indicate the number of the newly observed and predicted line features, *C^i^* and *C^j^* are the respective covariance matrices of the line features. *C^i^* was calculated from the former process (Equation (14)), *C^j^* is calculated from the UKF prediction step, and $\chi_{\gamma ,d}^{2}$ is a number taken from a χ^2^ distribution with d = 2 degrees of freedom and probability level γ = 99% on which the hypothesis of pairing correctness is rejected [46]. Then, the pairs that satisfy both conditions, which is to say, that are less than $\chi_{\gamma ,d}^{2}$ and one-to-one matches, are accepted and retained for the calculation of the measurement innovation (the observed minus the predicted value) [35], which is used later to estimate the a posteriori state $\hat{X}(t+1)$. If the pair only satisfies the former condition, that is, if it shows the one-to-many match, it is considered incorrect and is neglected. The non-matched observations greater than $\chi_{\gamma ,d}^{2}$ are transformed to the global frame and added to the next iterations as new features [47].

### 2.6. Unscented Kalman Filter

**Figure 5:**
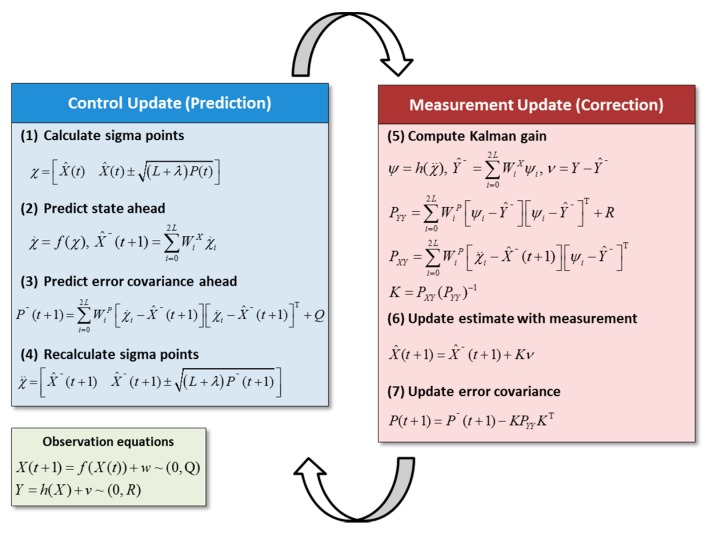
Process of Unscented Kalman filter.

The UKF is a variant of the Kalman filter which is specifically aimed at problems with nonlinear models, which not only gives better performance than that of the EKF, but also has several benefits in terms of ease of implementation. Its superior performances over that of the EKF algorithm have been reported in many SLAM studies [48,49,50,51,52]. Figure 5 describes the process of UKF SLAM. The mapping functions *f* and *h* represent the nonlinear, deterministic state and measurement models. The random variables *w* and *v* represent the process and measurement noise, and their noise covariance *Q* and *R* are assumed to be independent of each other, following the normal probability distributions, respectively. The UKF starts with the unscented transformation which computes the effect of a nonlinear function upon a mean *X* and covariance *P*. It operates by computing a deterministic sample set (sigma points) which is then propagated through the non-linearity [52]. In control update, *L* is the dimension of *X*, λ is the scaling parameter, and *W^X^* and *W^P^* are the weight for the *X* and *P*, respectively. Once the sigma points χ are obtained from the previous position $\hat{X}(t)$ and covariance *P*(*t*) in step (1), a current state (mean ${\hat{X}}^{-}(t+1)$ and covariance $P^{-}(t+1)$) is predicted in steps (2) and (3). Using the predicted mean and covariance, the sigma points are recalculated in step (4). In step (5) of measurement update, *Y* indicates newly observed features, and the predicted mean ${\hat{Y}}^{-}$ and covariance $P_{YY}$ and $P_{XY}$ of the measurement are calculated using the newly updated sigma points ψ for the measurement model h. Finally, the difference ν between the observed *Y* and the predicted features ${\hat{Y}}^{-}$ is multiplied by Kalman gain and used to correct the current system’s position in step (6) and covariance in step (7). For additional details, please see Thrun *et al*. [53], Andrade-Cetto, Vidal-Calleja and Sanfeliu [48], and Terejanu [54].

## 3. Experimental Results

### 3.1. Implementation of Kinematic Scanning System

**Figure 6:**
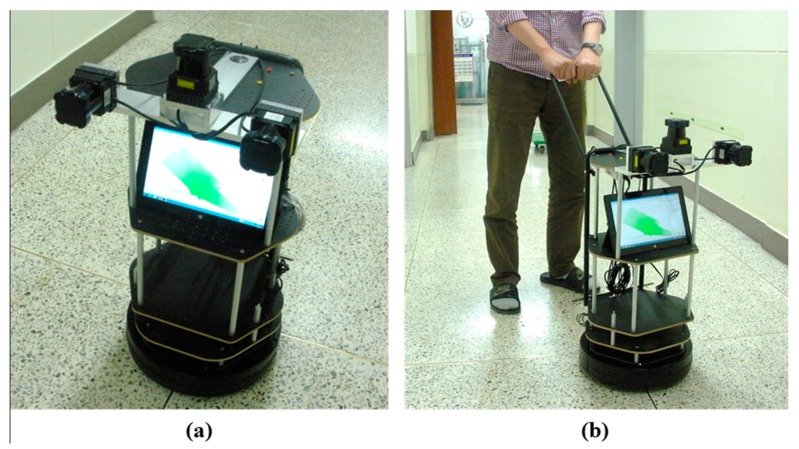
(**a**) Kinematic 3D laser scanning system and (**b**) system operation.

Figure 6a shows the kinematic 3D laser scanning system developed in our research. It measures approximately 35 cm (length) × 35 cm (width) × 78 cm (height). The platform is equipped with odometry, and carries a laptop computer (used for storing the data of each sensor) and three 2D laser range finders (Hokuyo UTM-30LX). The front laser range finder is mounted horizontally to map unknown environments and correct the position of the scanning system. The other two are mounted vertically to scan the profiles of surrounding environments while the scanning system moves. The 3D point cloud is obtained by registering those vertical profiles on the system’s trajectory. The scan area is 270° in the horizontal direction (with 1081 points) and 180° in the vertical direction (with 721 points), and the interval angle is 0.25°. This research assumes that the intrinsic sensor calibration is completed, and the extrinsic calibration process of the developed kinematic scanning system is given in Jung *et al*. [55] Since the current system is not designed for automatic navigation, the surveyor needs to manually move it in scanning an indoor space, as shown in Figure 6b.

**Figure 7:**
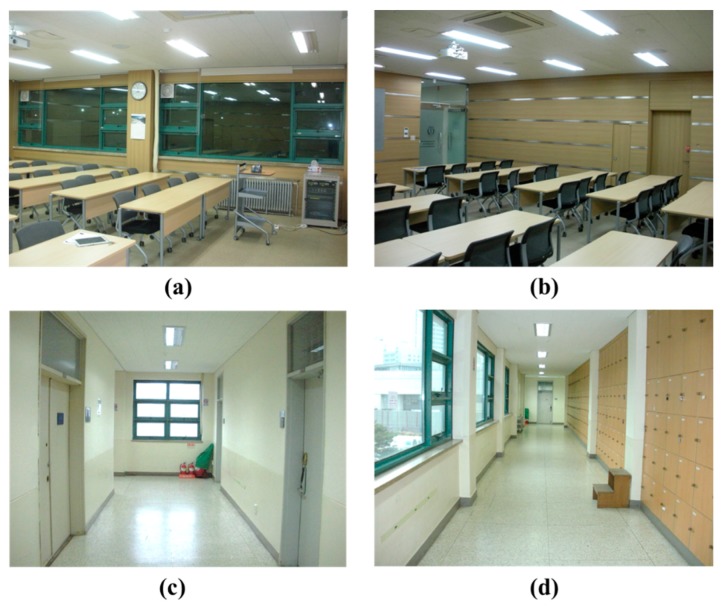
Test sites: (**a**,**b**) are the seminar room; (**c**,**d**) are the corridor.

A pilot implementation of the kinematic scanning system was conducted for two typical indoor places, a seminar room and a corridor at Yonsei University (Figure 7). The seminar room is a relatively small and simple structure including much clutter, whereas the corridor is longer and includes several pillars and a corner, though less clutter. The size of the seminar room is about 8.8 m (length) by 8.3 m (width), and the corridor is approximately 27.7 m long and 2.6 m wide.

For optimum filtering results, exact knowledge of the process and measurement noise covariance matrix (*Q* and *R*) is important. In practice, however, they are usually unknown and come from intensive empirical analysis [56,57]. To specify *Q* and *R*, one of the methods is to fix one of them and vary the other one by trial and error to find the smallest value that yields stable state estimates [58]. In this study, the diagonal element values of *Q* were determined to be (0.001 m)^2^ for x, y and (0.001 rad)^2^ for θ, and the diagonal element values of *R* were determined to be (0.019 rad)^2^ for ϕ and (0.024 m)^2^ for ρ.

In the experiment, three travels were performed for each test site in order to verify the effects of the proposed constrained SLAM approach. During the operation, if an obstacle was too far to be perceived, the feature detection process did not occur, causing the accumulation of odometry errors due to the absence of SLAM update feedback [59]. In practice, it was found that the line-feature extractions by the incremental algorithm with Hokuyo UTM-30LX increased linearly according to the threshold distance d (Figure 2); for example, if d was 0.01 m, line features up to about 10 m could be extracted, and 0.02 m was appropriate for 20 m. Because a too-large d for a small space extracts unnecessary line features, leading to computational complexity in the SLAM process, a proper threshold that takes due account of the size of the scan area should be adopted. Accordingly, in the present study, the threshold was determined to be 0.01 m for the small seminar room, and 0.02 m for the long corridor.

**Figure 8:**
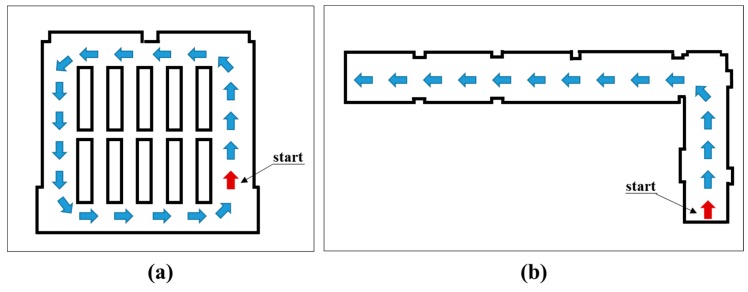
Trajectories of kinematic laser scanning: (**a**) the seminar room; (**b**) the corridor.

**Figure 9:**
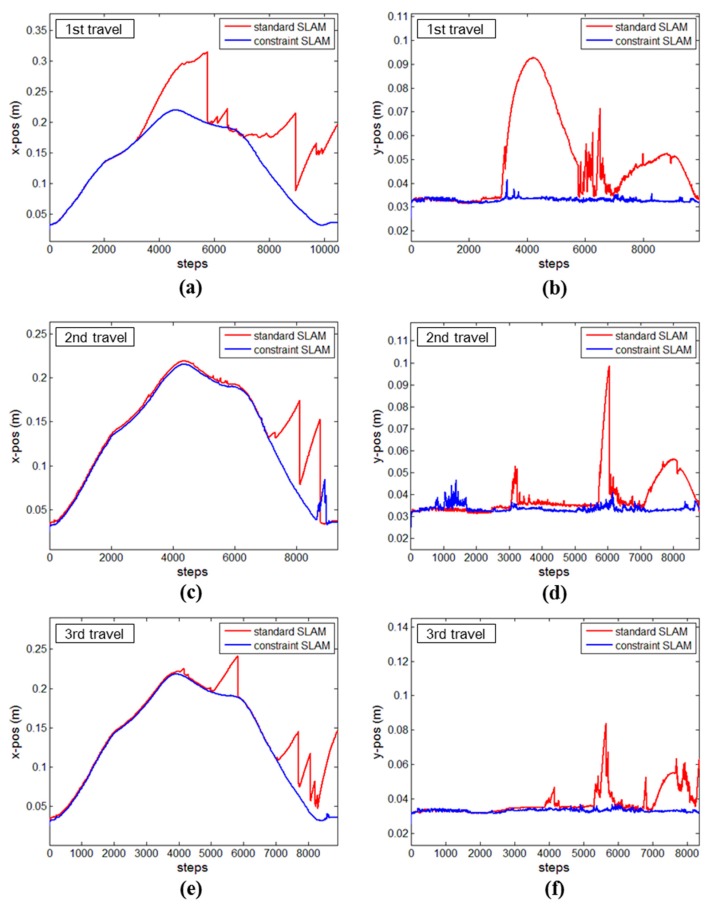
Uncertainty estimates of the seminar room test resulting from UKF SLAM without (red graphs) and with (blue graphs) the constrained approach: sideward direction (**a**,**c**,**e**); and forward direction (**b**,**d**,**f**).

Figure 8 shows the trajectories of the mobile system in two test sites. For the seminar room, the mobile system started to move at the lower-right corner, and traveled along the counter-clockwise path, revisiting the start point to obtain a complete map. Meanwhile, for the corridor, the mobile system started to move from the lower-right point and completed one-way travel. As the mobile system moved along the trajectory of the seminar room, its positional uncertainties in the sideward direction (Figure 9a,c,e) and the forward direction (Figure 9b,d,f) were recorded within two standard deviations (95% upper confidence level) [60]. In the figure, the red and blue graphs indicate the positional uncertainties of the standard SLAM and the constrained SLAM, respectively. Note that the mobile system was forced to turn sharply at the four corners. This generally would degrade the quality of navigation, since the odometry errors would quickly accumulate particularly for the sideward direction. Accordingly, in the first travel (Figure 9a), both the standard and constrained SLAMs show continuous increases of uncertainty until the mobile system reached the first turn (around 3000 time steps). After which, the standard SLAM shows drastic divergences of uncertainty, which were mainly due to failure in the data association phase, leading to loss of information for the correction in measurement update. Meanwhile, the constrained SLAM maintains smooth growth of uncertainty until the second turn (around 5000 time steps) and shows convergences, indicating that the system re-observed the line features in the beginning, which reduces the uncertainty for the line features as well as the system’s pose [53]. Similarly, in Figure 9c,e, the constrained SLAM maintains the consistent pattern, smooth convergences after divergences of uncertainty, whereas the standard SLAM shows abnormal divergences of uncertainty after half the time steps. The superiority of the constrained SLAM also can be found in the forward direction (Figure 9b,d,f): overall, the constrained SLAM successfully maintains the convergences during the entire time step, whereas the standard SLAM shows the drastic divergences of uncertainty again.

**Figure 10:**
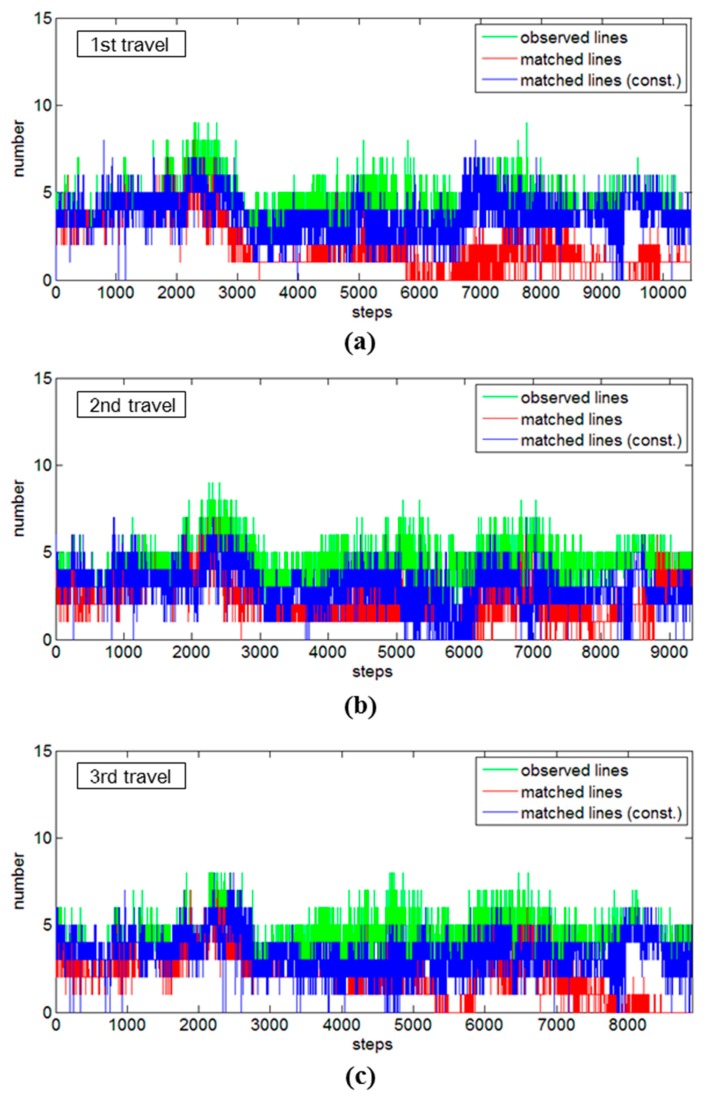
Comparisons of data association results for the seminar room test: (**a**) first travel; (**b**) second travel; and (**c**) third travel.

Figure 10 provides further qualitative evidence of the feasibility of the constrained approach. Note that the estimated mobile system’s trajectory is improved by matching newly observed features with previously stored features, thus it is desirable to obtain as much pairings as possible in the data association [38]. In the figure, the vertical axis denotes the number of features, and the horizontal axis represents the time steps. The green graph indicates the number of newly observed line features for each time step, and the red and blue graphs indicate the number of matched line features without and with the constrained approach, respectively. There are apparent discrepancies in the results without and with the constrained approach: all of the tests show that the constrained approach had higher matching numbers, commonly after the third turns (the red graph between 6000 and 9000 time steps in Figure 10), which resulted in the failures to maintain convergences of uncertainty for the standard SLAM in Figure 9. The overall matching rates in the seminar room test without and with the constrained approach were calculated as 45.4% and 85.8% for the first travel, 50.7% and 69.2% for the second travel, and 46.7% and 76.9% for the third travel, respectively. This result demonstrated the primary advantage of the proposed constrained approach for successive data association, as achieved by reducing the uncertainties of the adjusted line parameters and continuing to improve the localization accuracy.

**Figure 11:**
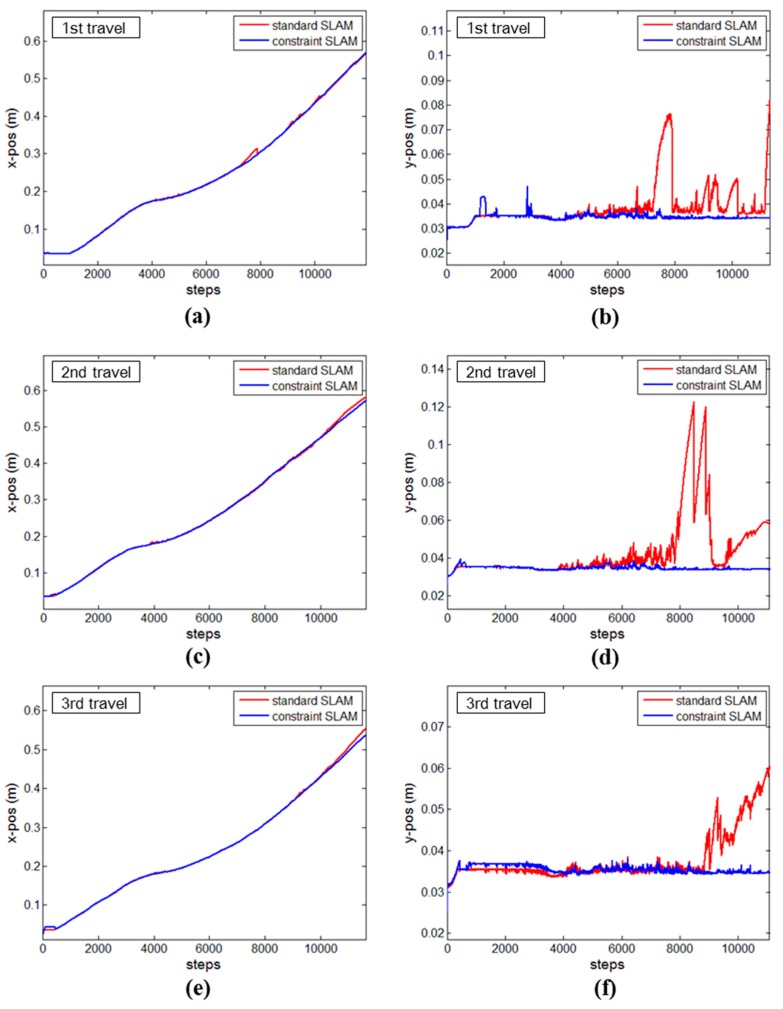
Uncertainty estimates of the corridor test resulting from UKF SLAM without (red graphs) and with (blue graphs) the constrained approach: sideward direction (**a**,**c**,**e**) and forward direction (**b**,**d**,**f**).

**Figure 12:**
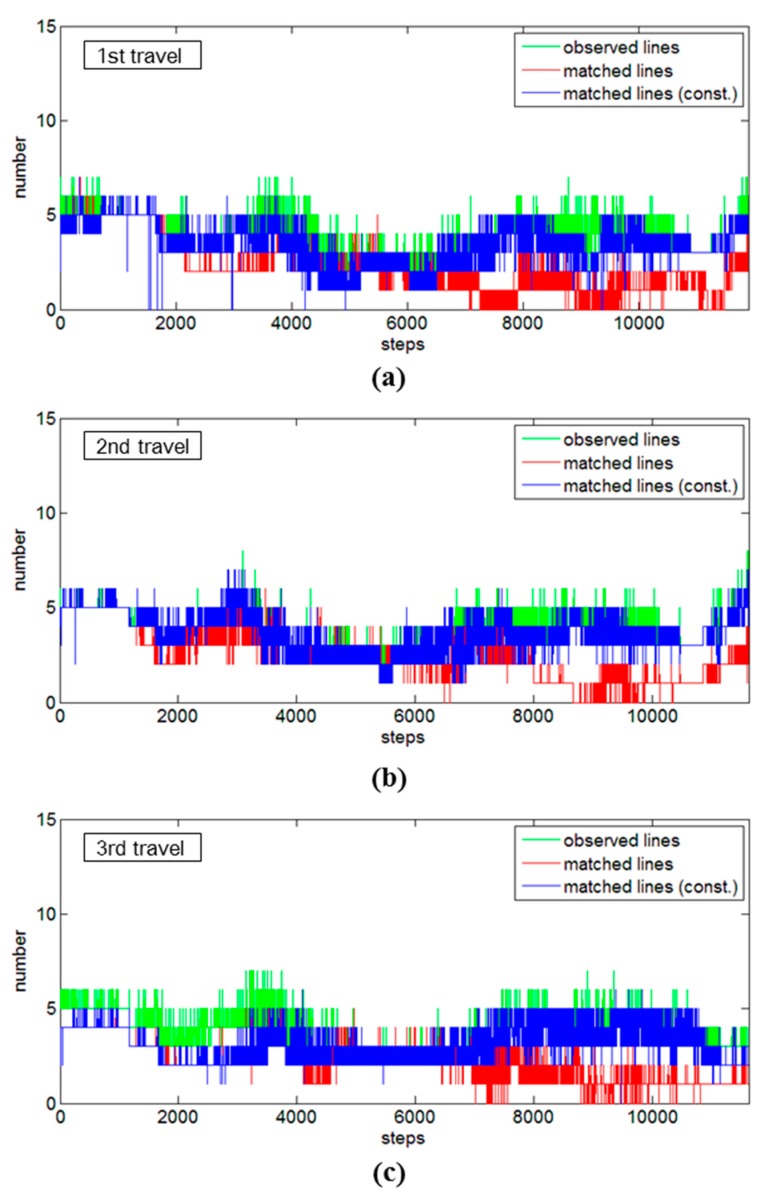
Comparisons of data association results for the corridor test: (**a**) first travel; (**b**) second travel; and (**c**) third travel.

The effects of the constrained approach were also found in the other test site, the corridor. This time, it completed one-way travel, thus all of the travels showed continuous growth of the system’s positional uncertainties in the sideward directions (Figure 11a,c,e). Compared with the seminar room tests, the differences between the standard SLAM and the constrained SLAM are not noticeable (possibly due to its straight and simple trajectory). As the system traveled along the trajectory, however, the standard SLAM started to show slightly larger divergences of uncertainty than the constrained SLAM. In fact, the difference is more noticeable in the forward directions (Figure 11b,d,f): after the mobile system reached half the time step, the standard SLAM shows the abnormal divergences of uncertainty. This can be explained also by the low matching numbers in the data associations in Figure 12, red graphs: the number of matched line features without the constrained approach (red graphs) gradually decreased, which lead to a lack of information and the abnormal divergences of system’s positional uncertainty. Satisfactory results, by contrast, could be achieved with the constrained SLAM: the application showed continuous convergences of uncertainty; likewise, the number of matched line features appeared to be well maintained at every time step (Figure 12, blue graphs). The overall matching rates in the corridor tests without and with the constrained approach were calculated as 55.8% and 88.0% for the first travel, 62.5% and 95.0% for the second travel, and 56.8% and 83.2% for the third travel, respectively.

### 3.2. Visualization of Point-Cloud Data

**Figure 13:**
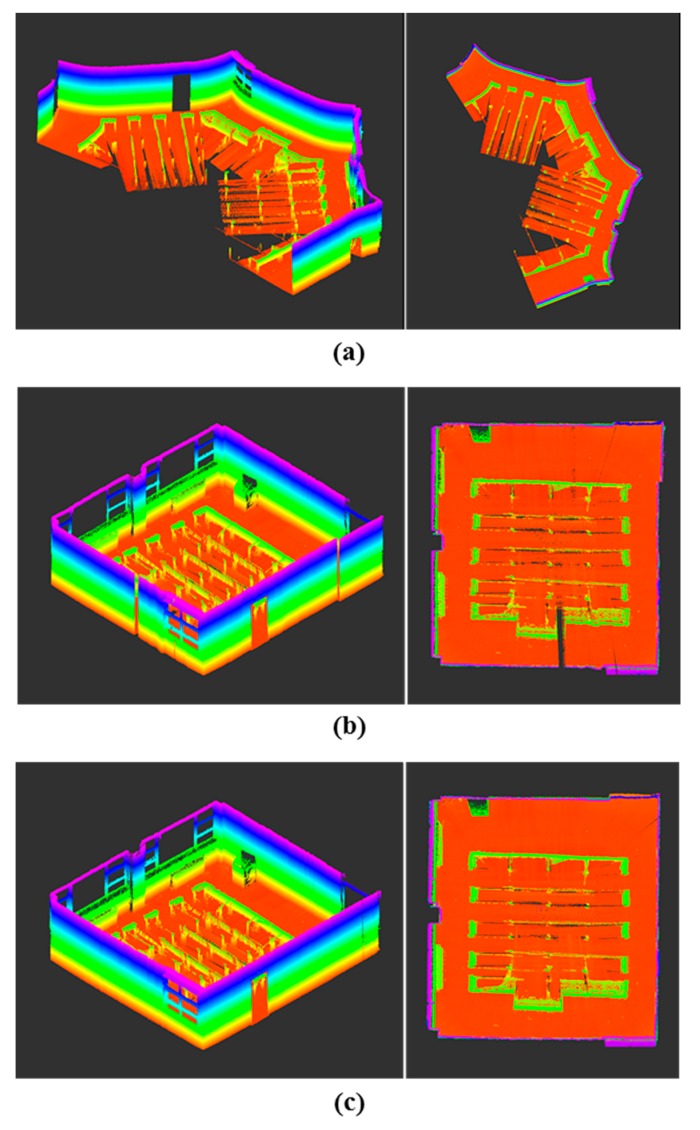
Point-cloud data acquisitions of the seminar room by kinematic scanning: (**a**) odometry only; UKF SLAM (**b**) without and (**c**) with the constraint approach.

**Figure 14:**
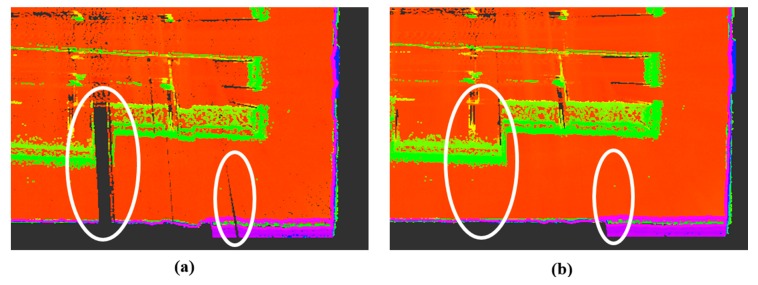
Detailed view of the seminar room (**a**) without and (**b**) with the constraint approach.

**Figure 15:**
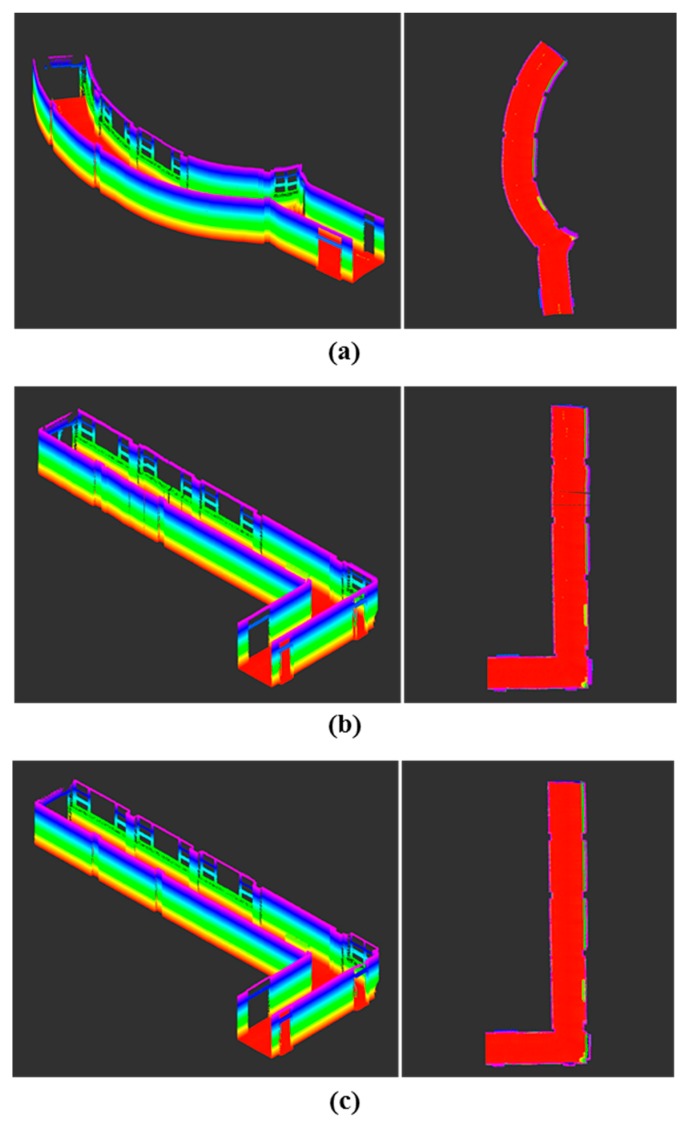
Point-cloud data acquisitions of the corridor by kinematic scanning: (**a**) odometry only; UKF SLAM (**b**) without and (**c**) with the constraint adjustment.

**Figure 16:**
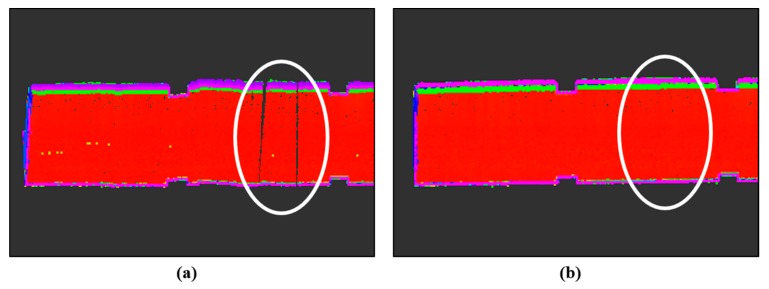
Detailed view of the corridor (**a**) without and (**b**) with the constraint approach.

The feasibility of the proposed constrained approach was further investigated with reference to the completeness of the mapping results. According to the highest line matching success rate, the first travel for the seminar room (85.8%) and the second travel for the corridor (95.0%) were selected. The first travel for the seminar room consisted of 9072 time steps (232.0 s) and resulted in a map with about 13.4 million points; the second travel for the corridor had 11,627 (324.8 s) time steps and yielded a map with about 20.2 million points. Note that the current scanning system provides complete observations of the surrounding environments; in Figure 13, Figure 14, Figure 15 and Figure 16, the ceiling points are omitted for the purposes of a clearer comparison.

A qualitative visual inspection of the seminar room revealed that the mapping accuracies with the odometry information only (Figure 13a) degraded quickly with distance traveled, due to the accumulated errors. In comparison, Figure 13b,c show that for the same scene, the mapping results from the use of the feature information were much more consistent. However, in the detailed view of the seminar room (Figure 14a,b), it is evident that the standard SLAM approach, showing a noticeable drift at the end, was not satisfactory (Figure 14a), whereas the mapping reconstruction by the constrained SLAM correspond more closely to the real environment (Figure 14b). Likewise, the visual inspection of the corridor results (Figure 15 and Figure 16) demonstrated the usefulness of the constrained approach for indoor mapping.

### 3.3. Accuracy Assessment

**Figure 17:**
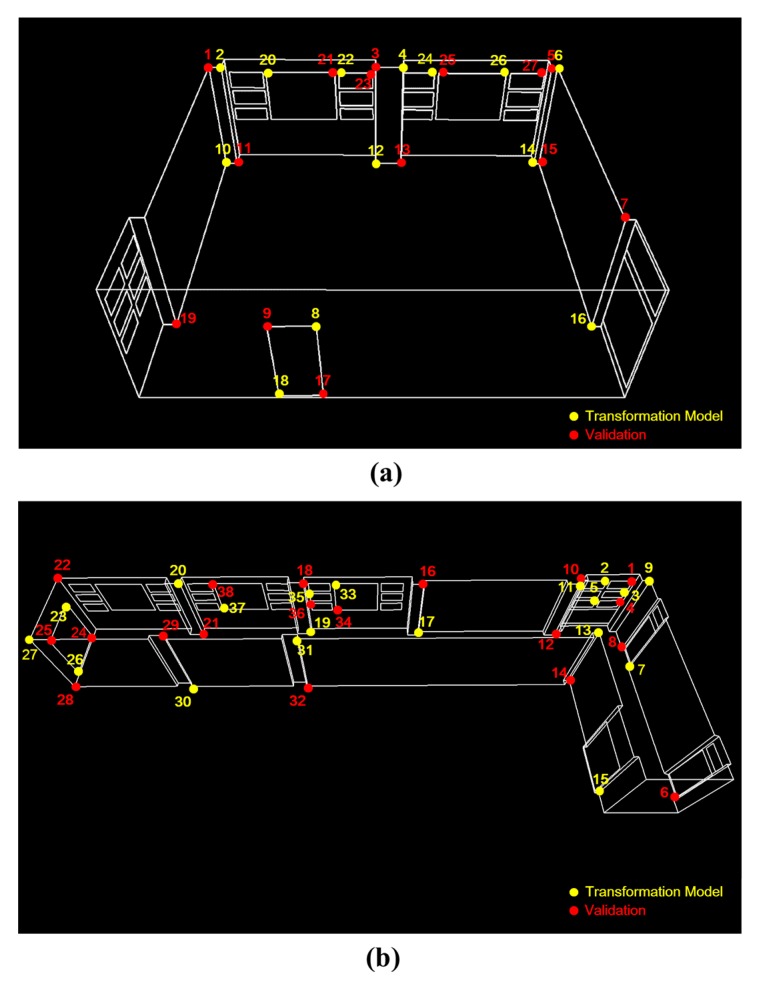
Check point distribution of (**a**) the seminar room; and (**b**) the corridor.

To test the feasibility and acceptability of the point-cloud acquisitions for the purpose of input to as-built BIM creation, the metric quality was assessed with the method proposed by Hong *et al.* [61] and the guidelines of the General Services Administration (GSA) for BIM accuracy [30]. The accuracy assessment was based on the well-distributed and clearly identifiable points such as corners of doors, walls, and windows as depicted in Figure 17. For the reference, the measurements acquired by a highly-accurate total station were used. First, the accuracy of the point-cloud data was assessed by means of the Euclidean average distance error ($\delta_{avg}$)

(17) $$\delta_{avg}=\frac{1}{n}\sum_{i=1}^{n}\left|\text{R}a_{i}-\text{T}-b_{i}\right|$$

where *a_i_* is the *i*-th check point in the point-cloud data, *b_i_* is the corresponding check point acquired by the total station, and R and T are the rotation and translation parameters for 3D Helmert transformation. Note that the scale was not considered in this transformation [62]. In Figure 17a, a total of 27 points were extracted from the seminar room, among which 14 were used to calculate the transformation model parameters (yellow points), and the remaining 13 for the validation (red points). For the corridor in Figure 17b, a total of 38 points were extracted: 19 for the transformation model (yellow points), and 19 for the validation (red points). The error vectors in the x, y, and z directions together with the corresponding average errors are listed in Table 1 (the seminar room) and Table 2 (the corridor). The Euclidean average distance error was calculated to 0.034 m for the seminar room and 0.043 m for the corridor, which satisfied the error tolerance (level 1) for point-cloud acquisition (0.051 m) according to the GSA guidelines [30]. Additionally, the quality of the point-cloud data was assessed by the Root Mean Square Error (RMSE) and the Spherical Accuracy Standard (SAS). The RMSE was calculated as

(18) $$RMSE=\sqrt{\frac{1}{n}\sum_{i=1}^{n}{\left(a_{i}^{t}-b_{i}\right)}^{2}}$$

where $a_{i}^{t}$ indicates the point transformed to the coordinates of the total station. The RMSEs for x, y, z directions also are listed in Table 1 and Table 2. The SAS, which represents the spherical radius of a 90% probability sphere [63], is defined as

(19) $$SAS=2.5\times 0.3333\times \left(RMSE_{x}+RMSE_{y}+RMSE_{z}\right)$$

The calculated SAS value was 0.050 m for the seminar room and 0.067 m for the corridor. This represents a positional accuracy of the two point-cloud acquisitions at the 90% confidence level. The main factors affecting the higher error for the corridor are: (1) one-way travel that did not allow for revisiting the starting point; and (2) the relatively small number of matched line features in the corridor (3.63 per time step) relative to that achieved for the seminar room (3.79 per time step).

**Table 1:** Accuracy assessment results for point-cloud data of the seminar room (unit: meter).

Point ID	Error Vector X	Error Vector Y	Error Vector Z	Error
1	−0.021	−0.013	0.005	0.025
3	−0.023	0.000	−0.013	0.027
5	0.003	−0.006	−0.015	0.016
7	0.010	0.061	0.021	0.066
9	0.030	−0.015	−0.013	0.036
11	−0.019	0.030	0.022	0.042
13	−0.021	0.013	0.002	0.025
15	0.018	0.001	0.017	0.025
17	0.022	−0.011	−0.001	0.025
19	0.031	0.009	−0.008	0.034
21	0.000	0.000	−0.004	0.004
23	0.007	−0.014	0.008	0.018
25	0.041	0.004	−0.001	0.041
27	0.029	−0.045	−0.002	0.053
Average error				0.034
RMSE	0.024	0.024	0.012	0.036
SAS				0.050

**Table 2:** Accuracy assessment results for point-cloud data of the corridor (unit: meter).

Point ID	Error Vector X	Error Vector Y	Error Vector Z	Error
1	−0.031	0.009	0.000	0.032
4	−0.059	0.019	−0.002	0.062
6	−0.062	0.023	0.022	0.069
8	−0.010	0.003	0.017	0.020
10	−0.013	−0.015	0.004	0.020
12	0.014	0.014	−0.034	0.039
14	−0.002	0.036	−0.039	0.053
16	0.011	0.025	0.011	0.030
18	0.007	−0.012	0.006	0.015
21	0.010	−0.024	0.007	0.026
22	0.008	−0.027	−0.007	0.029
24	0.016	−0.046	−0.011	0.050
25	0.056	−0.026	−0.002	0.062
28	−0.007	−0.033	−0.001	0.034
29	0.027	−0.010	−0.009	0.030
32	0.019	−0.025	−0.012	0.034
34	0.088	−0.022	−0.005	0.090
36	−0.002	−0.037	0.015	0.040
38	−0.063	−0.026	0.053	0.086
Average error				0.043
RMSE	0.036	0.025	0.019	0.048
SAS				0.067

## 4. Conclusions

The present study proposed a new line-feature-based SLAM technique incorporating the constrained least squares method for line adjustments. The superiority of the proposed approach, compared with the conventional methods, is its reduction of the adjusted lines’ uncertainties for successful data associations, which consequently leads to more accurate systems’ pose estimations. The experimental results showed accurate reconstructions of 3D scenes, demonstrating the proposed method’s potential utility for indoor mapping. Moreover, the proposed constrained adjustment method can be simply applied to any line-feature-based SLAM applications to indoor environments satisfying the parallel and orthogonal assumptions. 

In this light, we are currently looking into how this technique can be applicable to other feature-based methods. As regards feature extraction, interesting for the purposes of further research is the geometric constraints concept, which could make possible the implementation of new features (e.g., curves and circles) and their geometric relationships for various indoor conditions. Future work will also focus on combining new sensors such as camera and IMU (Inertial Measurement Unit) for improved navigation quality. The combined utilization of these sensors with laser scanners allows new sensor readings for redundancy, increasing localization performance and long-term stability. In addition, autonomous navigation for the developed kinematic scanning system is needed, because uncertainties arising from manual operation can lead to incorrect localization and mapping results. However, for the purposes of obtaining a complete map of complex and cluttered indoor environments, fully automated navigation is not practical at the present stage. The viable solution is semi-autonomous navigation such as marker-based SLAM, whereby the system’s location is identified with the marker attached to a surveyor or controlled by remote control. Ultimately, the proposed kinematic scanning system is applicable to the as-built BIM, where it can be used for fast and efficient raw point-cloud data acquisition. The next phase of the research will involve the automated recognition of objects from the point-cloud data, which should be followed to keep up with the requirements of SCAN-to-BIM conversion.

## References

[1] Turkan Y., Bosche F., Haas C.T., Haas R. Towards automated progress tracking of erection of concrete structures. Proceedings of the 6th International Conference on Innovation in Architecture, Engineering & Construction (AEC’10).

[2] Anil E.B., Akinci B., Huber D. Representation requirements of as-is building information models generated from laser scanned point cloud data. Proceedings of the International Symposium on Automation and Robotics in Construction (ISARC).

[3] Han S., Cho H., Kim S., Jung J., Heo J. (2012). Automated and efficient method for extraction of tunnel cross sections using terrestrial laser scanned data. J. Comput. Civil Eng..

[4] Randall T. (2011). Construction engineering requirements for integrating laser scanning technology and building information modeling. J. Construct. Eng. Manag..

[5] Heo J., Jeong S., Park H.K., Jung J., Han S., Hong S., Sohn H.G. (2013). Productive high-complexity 3D city modeling with point clouds collected from terrestrial lidar. Comput. Environ. Urban Syst..

[6] Tang P., Huber D., Akinci B., Lipman R., Lytle A. (2010). Automatic reconstruction of as-built building information models from laser-scanned point clouds: A review of related techniques. Autom. Construct..

[7] Jung J., Hong S., Jeong S., Kim S., Cho H., Hong S., Heo J. (2014). Productive modeling for development of as-built bim of existing indoor structures. Autom. Construct..

[8] Volk R., Stengel J., Schultmann F. (2014). Building information modeling (BIM) for existing buildings—Literature review and future needs. Autom. Construct..

[9] Pătrăucean V., Armeni I., Nahangi M., Yeung J., Brilakis I., Haas C. (2015). State of research in automatic as-built modelling. Adv. Eng. Inf..

[10] Becerik-Gerber B., Jazizadeh F., Kavulya G., Calis G. (2011). Assessment of target types and layouts in 3D laser scanning for registration accuracy. Autom. Construct..

[11] Arayici Y. (2008). Towards building information modelling for existing structures. Struct. Surv..

[12] Campbell D., Whitty M., Lim S. Mobile 3D indoor mapping using the continuous normal distributions transform. Proceedings of the 2012 International Conference on Indoor Positioning and Indoor Navigation (IPIN).

[13] Hähnel D., Burgard W., Thrun S. (2003). Learning compact 3D models of indoor and outdoor environments with a mobile robot. Robot. Auton. Syst..

[14] Weingarten J., Gruener G., Siegwart R. A fast and robust 3D feature extraction algorithm for structured environment reconstruction. Proceediings of the 11th International Conference on Advanced Robotics (ICAR).

[15] Nuchter A., Surmann H., Hertzberg J. Automatic model refinement for 3D reconstruction with mobile robots. Proceedings of the 2003 IEEE 4th International Conference on 3D Digital Imaging and Modeling (3DIM).

[16] Wang Y., Huo J., Wang X. A real-time robotic indoor 3D mapping system using duel 2D laser range finders. Proceedings of the 2014 33rd Chinese Control Conference (CCC).

[17] Yan R.J., Wu J., Lee J.Y., Han C.S. 3D point cloud map construction based on line segments with two mutually perpendicular laser sensors. Proceedings of the International Conference on Control, Automation and Systems (ICCAS), Kimdaejung Convention Center.

[18] Folkesson J., Christensen H. Graphical SLAM-A self-correcting map. Proceedings of the 2004 IEEE International Conference on Robotics and Automation (ICRA).

[19] Arras K.O., Castellanos J.A., Schilt M., Siegwart R. (2003). Feature-based multi-hypothesis localization and tracking using geometric constraints. Robot. Auton. Syst..

[20] Lee K.W., Wijesoma S., Guzmán J.I. (2007). A constrained SLAM approach to robust and accurate localisation of autonomous ground vehicles. Robot. Auton. Syst..

[21] Zunino G. Simultaneous localization and mapping for navigation in realistic environments. https://www.nada.kth.se/utbildning/forsk.utb/avhandlingar/lic/020220.pdf.

[22] Nguyen V., Harati A., Martinelli A., Siegwart R., Tomatis N. Orthogonal SLAM: A step toward lightweight indoor autonomous navigation. Proceedings of the 2006 IEEE/RSJ International Conference on Intelligent Robots and Systems (IROS).

[23] Choi Y.H., Lee T.K., Oh S.Y. (2008). A line feature based SLAM with low grade range sensors using geometric constraints and active exploration for mobile robot. Auton. Robots.

[24] Kuo B.W., Chang H.H., Chen Y.C., Huang S.Y. (2011). A light-and-fast SLAM algorithm for robots in indoor environments using line segment map. J. Robot..

[25] Choi H., Kim R., Kim E. (2014). An efficient ceiling-view SLAM using relational constraints between landmarks. Int. J. Adv. Robot. Syst..

[26] Wolf P.R., Ghilani C.D. (1997). Adjustment Computations: Statistics and Least Squares in Surveying and GIS.

[27] Snow K. (2009). Adjustment Computation Notes.

[28] Weingarten J. (2006). Feature-Based 3D SLAM.

[29] Wang H., Fu G., Li J., Yan Z., Bian X. (2013). An adaptive UKF based SLAM method for unmanned underwater vehicle. Math. Probl. Eng..

[30] General Services Administration (2009). Bim Guide for 3D Imaging, ver. 1.0.

[31] Garulli A., Giannitrapani A., Rossi A., Vicino A. Mobile robot SLAM for line-based environment representation. Proceedings of the 44th IEEE Conference on Decision and Control, and 2005 European Control Conference (CDC-ECC).

[32] An S.Y., Kang J.G., Lee L.K., Oh S.Y. SLAM with salient line feature extraction in indoor environments. Proceedings of the 2010 11th International Conference on Control Automation Robotics & Vision (ICARCV).

[33] Yap T.N., Shelton C.R. SLAM in large indoor environments with low-cost, noisy, and sparse sonars. Proceedings of the IEEE International Conference on Robotics and Automation, ICRA’09.

[34] Kaess M., Ranganathan A., Dellaert F. (2008). ISAM: Incremental smoothing and mapping. IEEE Trans. Robot..

[35] Welch G., Bishop G. (2006). An Introduction to the Kalman Filter.

[36] Pinto M., Moreira A.P., Matos A., Sobreira H. (2012). Novel 3D matching self-localisation algorithm. Int. J. Adv. Eng. Technol..

[37] Dissanayake G., Huang S., Wang Z., Ranasinghe R. A review of recent developments in simultaneous localization and mapping. Proceedings of the 2011 6th IEEE International Conference on Industrial and Information Systems (ICIIS).

[38] Castellanos J.A., Montiel J., Neira J., Tardós J.D. (1999). The spmap: A probabilistic framework for simultaneous localization and map building. IEEE Trans. Robot. Autom..

[39] Riisgaard S., Blas M.R. (2003). SLAM for dummies. Tutor. Approach Simul. Localiz. Mapp..

[40] Navarro D., Benet G., Martínez M. Line based robot localization using a rotary sonar. Proceedings of the IEEE Conference on Emerging Technologies and Factory Automation (ETFA).

[41] Zhang X., Rad A.B., Wong Y.K. (2008). A robust regression model for simultaneous localization and mapping in autonomous mobile robot. J. Intell. Robot. Syst..

[42] Nguyen V., Gächter S., Martinelli A., Tomatis N., Siegwart R. (2007). A comparison of line extraction algorithms using 2D range data for indoor mobile robotics. Auton. Robots.

[43] Siadat A., Kaske A., Klausmann S., Dufaut M., Husson R. An optimized segmentation method for a 2D laser-scanner applied to mobile robot navigation. Proceedings of the 3rd IFAC Symposium on Intelligent Components and Instruments for Control Applications.

[44] Mikhail E.M., Ackermann F.E. (1976). Observations and Least Squares.

[45] Ryu K.J. (2012). Autonomous Robotic Strategies for Urban Search and Rescue.

[46] Arras K.O., Tomatis N., Jensen B.T., Siegwart R. (2001). Multisensor on-the-fly localization: Precision and reliability for applications. Robot. Auton. Syst..

[47] Weingarten J., Siegwart R. Ekf-based 3D SLAM for structured environment reconstruction. Proceedings of the IEEE/RSJ International Conference on Intelligent Robots and Systems (IROS).

[48] Andrade-Cetto J., Vidal-Calleja T., Sanfeliu A. Unscented transformation of vehicle states in SLAM. Proceedings of the IEEE International Conference on Robotics and Automation (ICRA).

[49] Kim C., Sakthivel R., Chung W.K. (2008). Unscented fastslam: A robust and efficient solution to the SLAM problem. IEEE Trans. Robot..

[50] Martinez-Cantin R., Castellanos J.A. Unscented SLAM for large-scale outdoor environments. Proceedings of the IEEE/RSJ International Conference on Intelligent Robots and Systems (IROS).

[51] Shojaie K., Shahri A.M. Iterated unscented SLAM algorithm for navigation of an autonomous mobile robot. Proceedings of the IEEE/RSJ International Conference on Intelligent Robots and Systems (IROS).

[52] Chekhlov D., Pupilli M., Mayol-Cuevas W., Calway A. (2006). Real-time and robust monocular SLAM using predictive multi-resolution descriptors. Advances in Visual Computing.

[53] Thrun S., Burgard W., Fox D. (2005). Probabilistic Robotics.

[54] Terejanu G.A. (2009). Unscented kalman filter tutorial. Workshop on Large-Scale Quantification of Uncertainty.

[55] Jung J., Kim J., Yoon S., Kim S., Cho H., Kim C., Heo J. (2015). Bore-sight calibration of multiple laser range finders for kinematic 3D laser scanning systems. Sensors.

[56] Yu K.K., Watson N.R., Arrillaga J. (2005). An adaptive kalman filter for dynamic harmonic state estimation and harmonic injection tracking. IEEE Trans. Power Deliv..

[57] Ding W., Wang J., Rizos C., Kinlyside D. (2007). Improving adaptive kalman estimation in GPS/INS integration. J. Navig..

[58] Almagbile A., Wang J., Ding W. (2010). Evaluating the performances of adaptive kalman filter methods in gps/ins integration. J. Glob. Position. Syst..

[59] Abrate F., Bona B., Indri M. Experimental EKF-based SLAM for mini-rovers with IR sensors only. Proceedings of the 3rd European Conference on Mobile Robots (ECMR).

[60] Yang S.W., Wang C.C., Chang C.H. Ransac matching: Simultaneous registration and segmentation. Proceedings of the IEEE International Conference on Robotics and Automation (ICRA).

[61] Hong S., Jung J., Kim S., Cho H., Lee J., Heo J. (2015). Semi-automated approach to indoor mapping for 3D as-built building information modeling. Comput. Environ. Urban Syst..

[62] Reit B. (1998). The 7-parameter transformation to a horizontal geodetic datum. Survey Rev..

[63] Greenwalt C., Schultz M. (1968). Principles of Error Theory and Cartographic Applications.

